# _D_-Serine reflects kidney function and diseases

**DOI:** 10.1038/s41598-019-41608-0

**Published:** 2019-03-25

**Authors:** Atsushi Hesaka, Shinsuke Sakai, Kenji Hamase, Tatsuhiko Ikeda, Rakan Matsui, Masashi Mita, Masaru Horio, Yoshitaka Isaka, Tomonori Kimura

**Affiliations:** 1 KAGAMI Project, National Institute of Biomedical Innovation, Health and Nutrition (NIBIOHN), Osaka, Japan; 2 Reverse Translational Project, Center for Rare Disease Research, National Institute of Biomedical Innovation, Health and Nutrition (NIBIOHN), Osaka, Japan; 3 Department of Nephrology, Osaka University Graduate School of Medicine, Osaka, Japan; 4 Graduate School of Pharmaceutical Sciences, Kyushu University, Fukuoka, Japan; 5 KAGAMI LAB., Incubation Center, Shiseido Co., Ltd., Tokyo, Japan; 6 Department of Functional Diagnostic Science, Osaka University Graduate School of Medicine, Osaka, Japan

## Abstract

_D_-Amino acids, long-term undetected enantiomers of _L_-amino acids, are now emerging as potential biomarkers, especially for kidney diseases. Management of chronic kidney disease (CKD), a global problem with its high prevalence and poor prognosis, is currently unsatisfactory due to the difficulty in estimating kidney function and in early detection of diseases. We now show that intra-body dynamics of _D_-serine reflect kidney function and diseases. The blood level of _D_-serine correlated well with the actual glomerular filtration ratio, a key kidney function. This correlation was compatible with those of conventional kidney markers, and blood level of _D_-serine was relatively unaffected by such clinical factors as body size. The balance between excretion and reabsorption of amino acids by the kidney was controlled with chiral selectivity, and the reabsorption of _D_-serine was sensitive to the presence of CKD. The combination of blood level and urinary dynamics of _D_-serine effectively distinguished CKD from non-CKD. These lines of evidence provide new insights into the enantioselective amino acid dynamics in the human body that reflect disease pathophysiology. _D_-Serine may serve as a vital biomarker that suppress CKD onset through the precise assessment of kidney function and the diagnosis of CKD.

## Introduction

Ever since the discovery of _D_-amino acid oxidase in the kidney^1^, the physiological and pathological functions of _D_-amino acids in the human body have not been fully elucidated^2^. This is mainly due to the trace levels of _D_-amino acids in nature where their enantiomers, _L_-amino acids, are predominantly present. The presence of _D_-amino acids was sporadically reported in patients with kidney diseases^3,4^ until recently, when a study revealed the clinical significance of _D_-amino acids in chronic kidney diseases (CKD)^5^. CKD, mainly defined by a reduction in the glomerular filtration ratio (GFR), is a global medical problem with a high prevalence worldwide (over 850 million patients) and high morbidity and mortality^6,7^. Chiral metabolomics of CKD patients revealed that trace amounts of _D_-amino acids do exist in human blood, and that the blood levels of chiral amino acids are associated with several clinical factors^5^. One of these close clinical relationships is the association between plasma _D_-serine and the creatinine-based estimated GFR (eGFR), a commonly-used surrogate marker for kidney function. Another important finding is that chiral amino acids are prognostic markers for CKD. The levels of some chiral amino acids effectively identified CKD patients at high risk for worsening kidney function. Chiral amino acids are now emerging as new biomarkers for diseases.

A key challenge in the management of CKD is the early detection of kidney diseases, because precise and reliable methods to estimate GFR are lacking. The golden standard of GFR is measured by inulin clearance (clearance of inulin, Cin); however, Cin is rarely used due to its methodological complexity. As replacements, eGFR, calculated from kidney markers, such as serum creatinine, and recently serum cystatin C, is widely used. eGFR has several limitations^8^. One problem is that the kidney markers currently available are affected by other factors, such as muscular mass^9^. Another problem is that eGFR shows variable performance in higher GFR ranges, the ranges that require precise estimation for the diagnosis of CKD. Because eGFR has these two major disadvantages, which can prevent the early detection of kidney disease, we wondered if chiral amino acids could be used to determine GFR more precisely. Here we investigated the potential of _D_-amino acids as biomarkers for kidney function and diseases.

## Results

### The plasma level of _D_-serine strongly correlates with GFR

**Figure 1:**
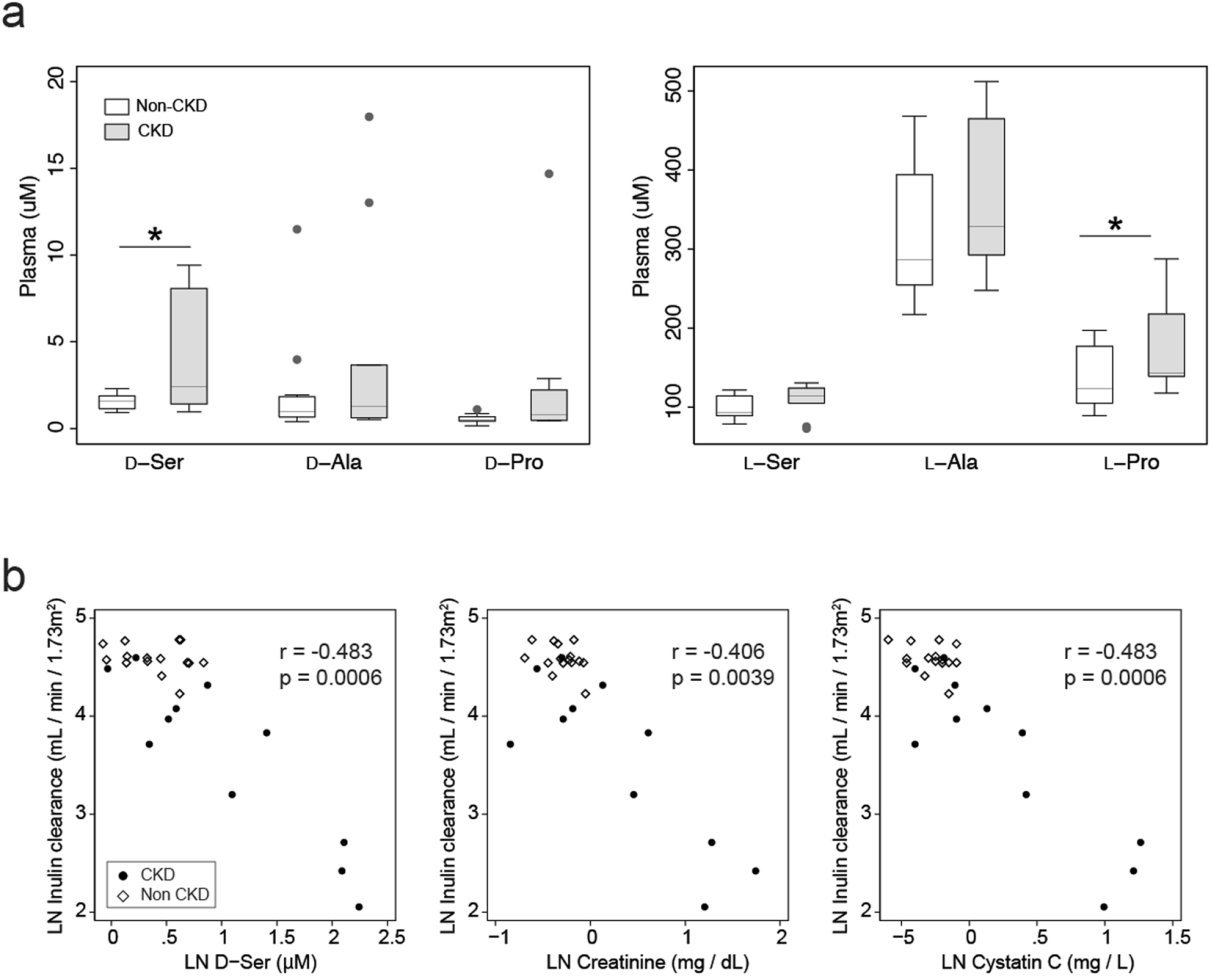
Relation of plasma _D_-serine and GFR. (**a**) Box plots of plasma levels of chiral amino acids. *P < 0.05 (Mann-Whitney *U*-test). (**b**) Blood levels of _D_-serine, creatinine, and cystatin C are plotted with GFR (mL/min/1.73 m^2^). Correlations, Kendall’s tau regression analyses. LN, log-natural transformed.

In order to examine the relationship between GFR and chiral amino acids, we measured inulin clearance, the golden standard for GFR, and simultaneously performed chiral amino metabolomics^10,11^. As a metabolomic platform, micro-two-dimensional high-performance liquid chromatography (2D-HPLC) was utilized to measure whole sets of _D_-amino acids with precision^10,11^. The first dimension of this system separated fluorescence-labelled amino acids by reverse-phase separation, followed by the second dimension of enantioselective separation. The plasma level of _D_-serine, previously shown to correlate with eGFR^5^, was higher in CKD patients than in non-CKD participants (Fig. 1a and Tables S1 and S2). Importantly, the plasma level of _D_-serine strongly correlated with the actual GFR (Fig. 1b). This correlation was compatible with those of conventional kidney markers (serum creatinine and cystatin C, Fig. 1b). Plasma levels of _D_-serine emerged as kidney markers that reflected GFR.

### Associations of _D_-serine plasma levels and clinical factors

To examine the intrinsic variation of chiral amino acids and their relationships with conventional kidney markers (creatinine and cystatin C), we performed principal component analysis (PCA)^12^. PCA geometrically projects complexed, higher-dimensional data onto lower dimensions called principal components. PCA of plasma chiral amino acids revealed that plasma _D_-amino acids, especially _D_-serine, showed a relatively similar profile to those of kidney markers (creatinine and cystatin C, Fig. S2), confirming that _D_-serine is a kidney marker.

Conventional kidney markers are known to be affected by such clinical factors as body size, age and sex^8,9^. The CKD of older individuals and/or individuals with muscle wasting (sarcopenic individuals) has been overlooked today because reduced muscular mass of these populations decreases the levels of creatinine, a waste product of muscular metabolism, and GFR of these individuals has been overestimated. In order to estimate the effects of clinical factors on the plasma level of _D_-serine, we performed orthogonal projection to latent structures (OPLS) analysis. OPLS analysis is a multivariate method used to identify predictive factors for a certain variable that separate the variation into target correlating variation (p1) and target non-correlating variation (pOrtho1). The OPLS model based on the plasma level of _D_-serine showed that kidney markers and inulin clearance were strong predictors, and age was a marginal predictor for plasma _D_-serine, whereas body size, as represented by body surface area (BSA), and sex were uncorrelated factors (Fig. S3). This OPLS model had both good explainability and predictive performance, as judged by score plot and high values of R^2^Y and Q^2^Y (Fig. S3). Indeed, the plasma level of _D_-serine was relatively unaffected by BSA, while kidney markers showed correlations with BSA in participants with relatively good kidney function as reported previously^9^ (Supplementary Fig. S4a,b).

As GFR decreases with age, age correlates with inulin clearance^13^. Because our studied population contained aged CKD patients, the plasma level of _D_-serine marginally correlated with age as did inulin clearance; however, there was no correlation between the _D_-serine plasma level and age when participants with relatively good kidney function were assessed (Supplementary Fig. S5a,b). Thus, _D_-serine appeared to be unrelated with age, at least in the population with better GFR. In summary, the plasma level of _D_-serine was a marker of GFR irrespective of clinical factors such as body size, age and sex.

### Urinary excretions of amino acids are chiral selective

The key regulator of intra-body chiral amino acid distribution is urinary excretion by the kidney^11,14^. The urinary ratios of _D_-amino acids per total amino acids (%D) in some amino acids were much higher than blood ones (Tables S2, S3 and Supplementary Fig. S6). In non-CKD population, for example, the %D of _D_-serine was 45.4% in urine whereas it was 1.46% in blood (Tables S2, S3 and Supplementary Fig. S6). Interestingly, _D_-glutamine, _D_-threonine, _D_-methionine, _D_-valine and _D_-isoleucine, which have never been detected in human body^5^, were observed in the urine. These findings corroborate the concept that _D_-amino acids are present in the human body and are excreted via the kidney^5^.

**Figure 2:**
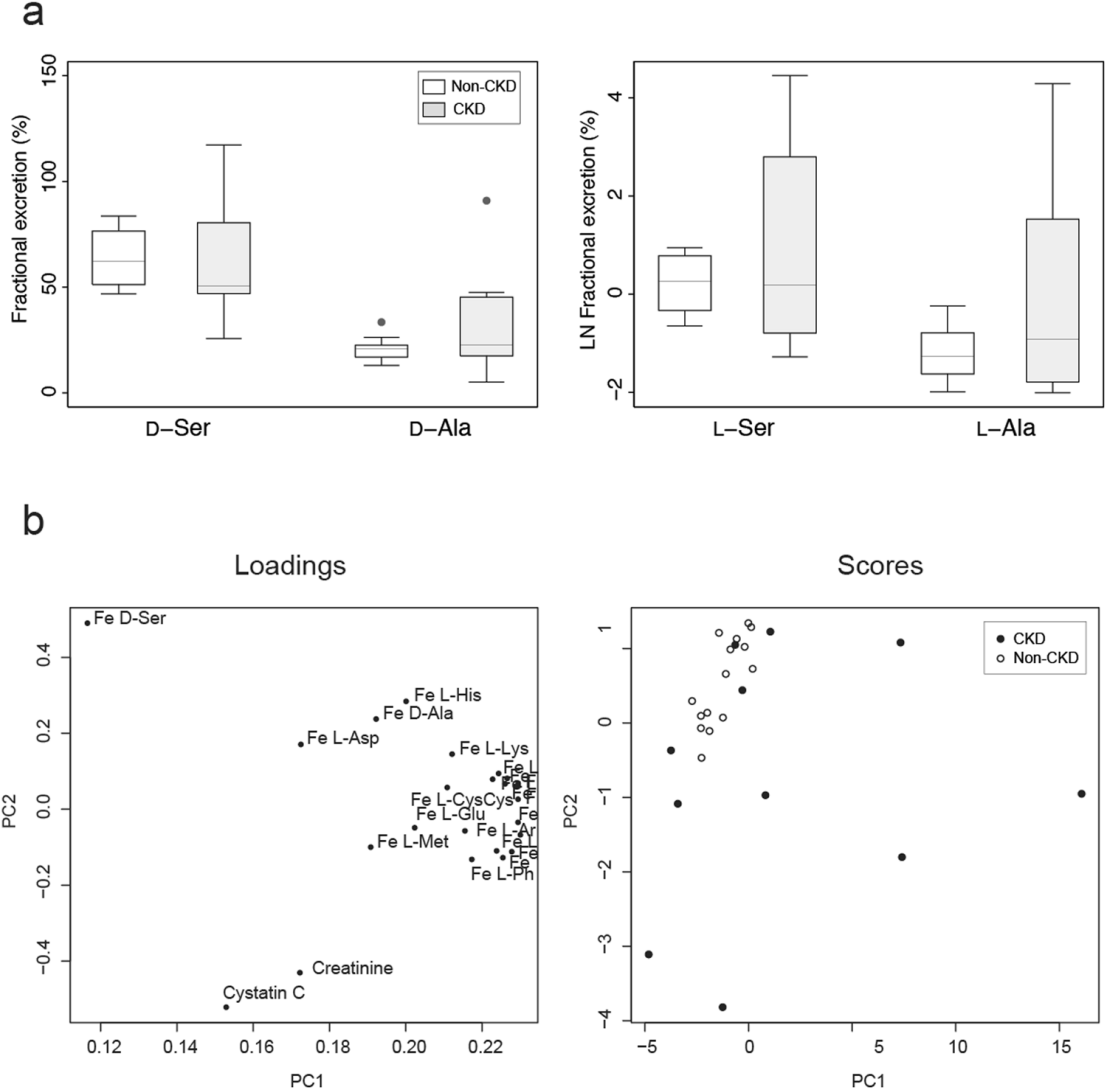
Characteristics of the urinary chiral amino acid profile. (**a**) Box plots of fractional excretions (Fe) of chiral amino acids. (**b**) Primary component analysis (PCA) of Fe of chiral amino acids, and serum levels of creatinine and cystatin C. The score value of each observation is plotted on the score plot to demonstrate the clusters of observations. Of note, Fe of _L_-amino acids were densely constricted within the 3 o’clock direction of the loading plot.

To assess the urinary excretion dynamics of chiral amino acids, we calculated fractional excretions (Fe) of the chiral amino acids. Fe is the ratio of a substrate filtered by the kidney glomeruli that is excreted into the urine. Fe represents kidney tubular handlings (reabsorption or secretion) of a substrate. Fe of _L_-amino acids, for example, are known to be maintained at very low levels (between 0.2% and 2.5%) by tubular tight reabsorption; nearly 99% of filtered _L_-amino acids are reabsorbed by the proximal tubule^15^. Fe of _D_-serine (62.1 [53.4–73.6] %) and _D_-alanine (20.7 [17.7–22.2] %) in healthy participants were much higher than those of enantiomer counterparts (Fig. 2a and Table S4), but the variances were maintained relatively low within certain ranges.

PCA confirmed the distinct profiles of Fe of _D_-amino acids (Fig. 2b). While the Fe of _L_-amino acids were closely constricted to one area (3 o’clock area in the loading plot, Fig. 2b) due to the similar modes of reabsorption, Fe of _D_-serine showed completely different profiles from kidney markers and Fe of _L_-amino acids. Kidney tubules were shown to handle the urinary excretion dynamics of _D_-amino acids by modes that were different from those of _L_-enantiomers.

### _D_-Serine dynamics are strongly associated with disease profiles

**Figure 3:**
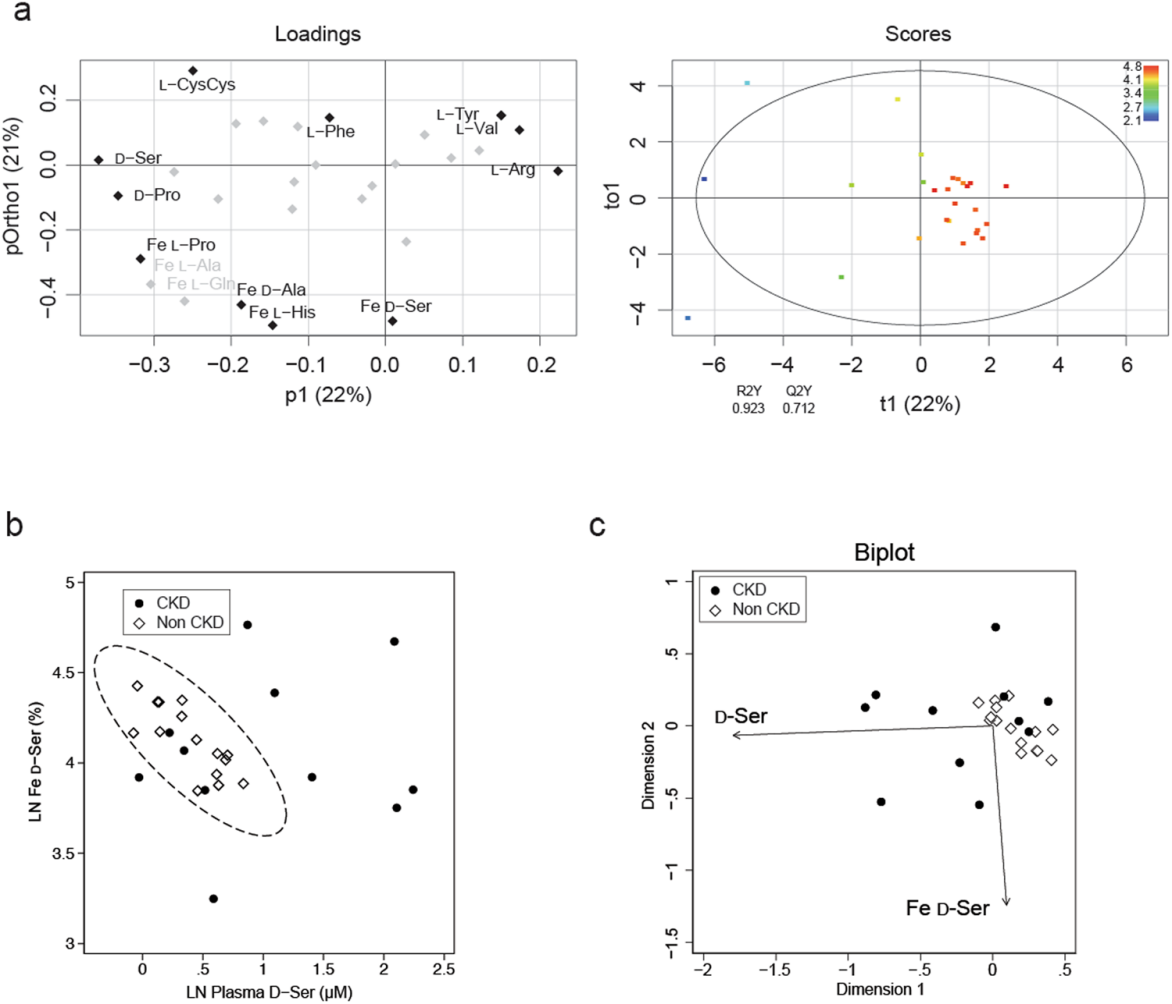
Relation of _D_-serine dynamics and disease profile. (**a**) OPLS derived from the chiral amino acid profile, and serum levels of creatinine and cystatin C, on GFR. Fe of _L_-amino acids used in this model are indicated. The remaining gray symbols represent plasma _L_-amino acids. The score plot is colored according to LN GFR. The circle in the score plot represents the 95% confidence interval. (**b**) Relation between the _D_-serine profile and CKD. The eclipse represents 95% confidence interval of non-CKD population. (**c**) A biplot visualization of the _D_-serine profile for CKD.

Then, how are plasma and Fe of _D_-amino acids interconnected? To evaluate the diverse mechanisms of kidney handling of each amino acid, we performed OPLS analyses using plasma levels and Fe of amino acids based on GFR. To avoid overfitting, some representative Fe of _L_-amino acids were selectively included in this model based on the very similar profile observed in the PCA analysis (Fig. 2b). As predicted, _D_-serine was classified as the strongest predictor of GFR; _D_-serine was on the further end of the p1 axis. However, Fe of _D_-serine was plotted on the vertical axis (pOrtho1) of OPLS (Fig. 3a), suggesting that Fe of _D_-serine is an uncorrelated factor of GFR and represents information other than GFR. The OPLS model had a good explainability of the variance of GFR, as judged by the R^2^Y value (0.923, Fig. 3a). This OPLS model also indicated that some amino acids are useful for the prediction of GFR variations. Indeed, some of them showed good to moderate correlations with GFR, although some of these associations were relatively limited in certain ranges of GFR levels (Fig. S7).

Given the emergence of chiral-selective clearances of amino acids by kidney, a question arouses if these regulatory mechanisms are pathologically related. To further explore the pathological information reflected by the Fe of _D_-serine, we plotted plasma levels and Fe of _D_-serine on a scatter plot. The dots of non-CKD participants were restricted to the bottom-left area (a plasma-low and Fe-middle profile, Fig. 3b). On the other hand, the profile of CKD patients was broad but was different from that of non-CKD participants (Fig. 3b). A biplot analysis was performed to graphically project the variables and the observations onto a two-dimensional principal component axes to simultaneously demonstrate the clusterings of observations and the correlations of the variables^16^. The biplot display showed that the combination of plasma and Fe values of _D_-serine effectively separated the CKD from non-CKD participants (Fig. 3c). Thus, the Fe of _D_-serine reflected the presence of CKD. These results also suggest that pathological kidneys handle chiral amino acids differently from those of healthy kidneys, and that the assessment of kidney handling of chiral amino acids can discriminate pathological conditions and have a strong diagnostic utility.

## Discussion

This study demonstrated the clinical utilities of chiral metabolomics, and provided a theoretical background for chiral amino acids as future biomarkers for CKD. The strong correlation with GFR identifies _D_-serine as a marker for GFR. Precise estimation of GFR in a healthy population is the key to detecting kidney disease as early as possible^6^, and _D_-serine will be useful for improved screening and management of CKD. The relatively independent nature of _D_-serine from body size is an advantage in the estimation of GFR. Additional information, such as age, sex, creatinine, and cystatin C for _D_-serine would help establish a new equation for the estimation of GFR. Moreover, the urinary dynamics of _D_-serine reflected the presence of CKD, and the combination of blood level and urinary dynamics of _D_-serine effectively separated CKD from non-CKD. Thus, _D_-serine serves as a dual biomarker, both for the estimation of kidney function and the detection of CKD and will facilitate comprehensive management of CKD. Nowadays, CKD is often overlooked when eGFR alone is measured^6^. _D_-Serine may provide additional information on eGFR that would benefit CKD patients for the diagnosis.

The excretion dynamics of _D_-amino acids are unique. The modes of urinary excretion of _D_-amino acids are absolutely different from those of _L_-amino acids. While nearly 100% of _L_-amino acids are reabsorbed by tubules, large portions of _D_-serine and _D_-alanine are not reabsorbed and are excreted into the urine. Less efficient uptake of _D_-serine than _L_-serine was also reported in animal kidney tubules using isotopes-labelled amino acids^17,18^. Additionally, such _D_-amino acids as _D_-asparagine and _D_-glutamine were shown to be excreted into urine excessively because, unlike their enantiomers, these _D_-amino acids were solely detected in urine but not in blood. The excretion dynamics of _D_-amino acids were also different from those of conventional kidney markers. Creatinine as a kidney biomarker is compromised by positive excretion. Cystatin C is presumed to be totally reabsorbed by tubules because only trace amounts of cystatin C are detected in urine^9^.

In spite of bulk excretion, the excretion of _D_-amino acids was regulated. The variances of Fe _D_-amino acids were restricted within certain ranges in non-CKD participants. Involvement of the _D_-amino acid reabsorption system based on transporters has been suggested^15,16^. These transporters, potentially either _D_-amino acid non-selective or selective, should be less effective than those of _L_-amino acids.

Interestingly, the reabsorption system of _D_-amino acids was sensitive to the presence of CKD. The variances in Fe of _D_-amino acids were broader in CKD patients. Of note, some patients showed increased excretion of _D_-serine (Fig. 3c, highlighted in magenta in Fig. S8). In these patients, the blood level of _D_-serine was almost normal or only slightly increased. Increased excretion of _D_-serine may be a compensatory mechanism to keep blood _D_-serine low. Another patient showed increased blood levels of _D_-serine with a decrease in Fe (Fig. 3c, highlighted in cyan in Fig. S8). The decreased reabsorption system of _D_-serine, affected by CKD, may cause an increase in the blood levels of _D_-serine. The increase and decrease in Fe of _D_-serine resulted in the broadness of Fe ranges in CKD patients, and resultantly, Fe _D_-serine alone was not significant between CKD and non-CKD (Fig. 2a).

In summary, the plasma level of _D_-serine increased in response to decreased urinary excretion due to reduced GFR. Kidneys with reduced GFR cannot increase the urinary excretions of _D_-serine compensatory, and resultantly, the blood level of _D_-serine may increase with the advancement of CKD. When we use 95% confidence eclipse based on plasma levels and Fe of _D_-serine from non-CKD population as a threshold, the sensitivity and specificity for detecting CKD were 72.7% and 100%, respectively (Fig. 3b). This good separation, which was not achieved either plasma _D_-serine or Fe of _D_-serine alone, suggests that the dynamics of _D_-serine, reflected by the combination of blood and urinary levels of _D_-serine, may represent kidney pathology and have a potential diagnostic utility in kidney diseases.

Our study has some limitations that need to be acknowledged. First, CKD patients studied in this study had relatively high GFR values, and further study is necessary to test patients with poor GFR levels. Second, the CKD population is heterogeneous, and the results presented here may need to be confirmed in specific type of kidney diseases. Some CKD patients with better GFR, as also estimated from GFR-correlating plasma _D_-ser axis, were within the 95% confidence eclipse of non-CKD participants  in Fig. 3b, and how to separate these patients from non-CKD population remains unsolved. Third, the number of participants was limited. These are worthy points to be validated in future studies to increase the significance of _D_-amino acids as biomarkers. Although the causal relationship remains to be solved, the fact that aberrant Fe of _D_-serine can discriminate pathological status has important implications for disease screening in clinics.

In conclusion, the levels of _D_-serine in blood and urine are regulated by kidneys, which are affected in the presence of CKD. The dynamics of _D_-serine reflect kidney function and diseases. The major findings reported herein, _D_-serine as a dual biomarker for kidney function and the presence of CKD, will contribute to better management of CKD. The important, but yet mysterious, relationship between the kidney and chiral amino acids will help elucidate unknown pathophysiology of various diseases.

## Methods

### Study population

We retrospectively enrolled consecutive 11 patients between 2016 and 2017 from a cohort consisted of CKD patients hospitalized to Department of Nephrology, Osaka University Hospital for diagnosis and/or treatment purpose. The origin of kidney diseases included: diabetic nephropathy (n = 1), IgA nephritis (n = 2), Fabry’s disease (n = 1), autosomal dominant polycystic kidney disease (n = 1), minimal change nephritic syndrome (n = 1), tubulointerstitial nephritis (n = 1), myeloma-related kidney disease (n = 1), primary aldosteronism (n = 2), benign prostatic hyperplasia (n = 1). Separately, fifteen non-CKD volunteers ages 20 years or older were also recruited at National Institute of Biomedical Innovation, Health and Nutrition (NIBIOHN). The study protocols were approved by the Ethical Review Board of Osaka University Hospital and by the Ethical Committee of NIBIOHN. This study was conducted in compliance with the ethical principles of the Declaration of Helsinki, and all participants gave written informed consent.

### Methods of inulin kidney clearance

Inulin clearance (clearance of inulin, Cin) was calculated from serum and urine inulin concentrations and urine volume using standardized methods described previously^19^. In brief, three sets of serum and urine samples were collected at three different time points during the 2-hour continuous intravenous infusion of 1% inulin (Fuji Yakuhin Co. Ltd.) given under fasting, medication-suspended, and hydrated condition. The participants received 500 ml of water orally 30 minutes before the infusion. In order to maintain hydration, 60 ml of water was given at 30, 60 and 90 minutes after the start of inulin infusion. The initial rate of infusion was 300 mL/h for the first 30 minutes, followed by 100 mL/min for 90 minutes. Blood samples were collected at 45, 75 and 105 minutes after the initiation of inulin infusion. Participants were instructed to empty the bladder completely at 30 minutes after initiation of infusion. Then, urine samples were collected between 30 and 60 minutes, between 60 and 90 minutes, and between 90 and 120 minutes. The inulin samples were measured using enzymatic methods. The mean of the three Cin values was used as the Cin by standard method (Cin-ST). Cin [mL/min] was calculated from total urinary inulin excretion per time [mg/min] divided by blood levels of inulin [mg/mL]. Blood inulin is filtrated in the kidney glomerular without reabsorption by tubules, and thus, Cin is used as a golden standard of glomerular filtration ratio.

For CKD patients, inulin clearance was calculated using a simplified single urine collection method as described previously^19^. Briefly, blood samples were collected at 45 minute after the initiation of inulin infusion. Then, participants were instructed to empty the bladder, and urine samples were collected for 60 minutes hereafter. Hydration and infusion were conducted in the same manners as done in standardized methods. Cin for non-CKD participants were all in a normal range (above 60 mL/min /1.73 m^2^), while Cin for CKD patients showed worse kidney function (Supplemental Table 1). Because single threshold for GFR above 60 mL/min /1.73 m^2^ is often insufficient in the real world, we tested some GFR thresholds as sensitivity analyses.

### GFR equations and kidney clearance calculation

Estimated GFR (eGFR) were calculated using the Japanese GFR equation based on serum creatinine (eGFR_creat_)^20^ and the Japanese GFR equation based on serum cystatin C (eGFR_cys_)^21^.

$${{\rm{eGFR}}}_{{\rm{creat}}}({\rm{mL}}/\,{\rm{\min }}\,/1.73\,{{\rm{m}}}^{2})=194\times {{\rm{Cr}}}^{-1.094}\times {{\rm{age}}}^{-0.287}\times 0.739\,({\rm{if}}\,{\rm{female}}).$$

$${{\rm{eGFR}}}_{{\rm{cys}}}({\rm{mL}}/\,{\rm{\min }}\,/1.73\,{{\rm{m}}}^{2})=(104\times {{\rm{Cys}}}^{-1.019}\times {0.996}^{{\rm{age}}}\times 0.929\,({\rm{if}}\,{\rm{female}}))-8.$$

Serum and urinary creatinine was measured enzymatically, and serum cystatin C was measured using gold colloid aggregation method. Spot urinary levels of chiral amino acids were adjusted by that of creatinine^22^.

Kidney clearance of substrates [mL/min] were calculated from total urinary substrates excretion per time [mg/min] divided by blood levels of substrates [mg/mL]. Kidney clearance reflects the excretion of substrates into the urine, the resultant of glomerular filtration and tubular reabsorption and/or secretion.

Fractional excretion (Fe, %) were calculated from clearance of substrate divided by that of creatinine, as follows.

$$\begin{array}{ccccc}Fe\,Substrate & = & \frac{Substrate\,clearance}{{\rm{Creatinine}}\,{\rm{clearance}}} & = & \frac{{\rm{Us}}\times {\rm{V}}/\mathrm{Ps}}{{\rm{Ucre}}\times {\rm{V}}/\mathrm{Pcre}}\\  &  & \,=\,\frac{{\rm{Us}}\times {\rm{Pcre}}}{{\rm{Ucre}}\times {\rm{Ps}}} &  & \end{array},$$

where Us and Ps represent urinary and plasma levels of substrate, respectively. Fractional excretion is the ratio of a substrate filtered by the kidney glomerular that is excreted in the urine. Low and high fractional excretion indicates tubular reabsorption and excretion, respectively.

### Sample preparation

Sample preparation from human plasma and urine was performed as previously described with modification^10,11^. In brief, 20-fold volumes of methanol were added to the sample and an aliquot (10 μL of the supernatant obtained from the methanol homogenate) was placed in a brown tube and used for NBD derivatization (0.5 μL of the plasma was used for the reaction). After drying the solution under reduced pressure, 20 μL of 200 mM sodium borate buffer (pH 8.0) and 5 μL of fluorescence labeling reagent (40 mM 4-fluoro-7-nitro-2,1,3-benzoxadiazole (NBD-F) in anhydrous MeCN) were added, then heated at 60 °C for 2 min. An aqueous 0.1% (v/v) TFA solution (75 μL) was added, and 2 μL of the reaction mixture was subjected to 2D-HPLC.

### Determination of amino acid enantiomers by 2D-HPLC

The enantiomers of amino acids were quantified using the micro 2D-HPLC platform, as previously described^10,11^. In brief, the NBD-derivatives of the amino acids were separated using a reversed-phase column (monolithic ODS column, 0.53 mm i.d. × 1000 mm; provided by Shiseido, Tokyo, Japan) with the gradient elution using aqueous mobile phases containing MeCN, THF, and TFA. In order to separately determine the _D_- and _L_-forms, the fractions of the target amino acids were automatically collected using a multi-loop valve, and transferred to the enantioselective column (KSAACSP-001S or Sumichiral OA-3200, 1.5 mm i.d. × 250 mm; self-packed. Materials were obtained from Shiseido and Sumika Chemical Analysis Service, Osaka, Japan, respectively). For the measurements of Ile and Thr, which has 4 stereoisomers (_L_-, _D_-, _L_-*allo*-, and _D_-*allo*-forms), we separated (_L_- and _D_-) forms and diastereomer (_L_-*allo*- and _D_-*allo*-) forms by the reversed-phase mode in the first dimension (these diastereomers are separable by the reversed-phase mode). Then their enantiomers (_L_- and _D_-, _L_-*allo*- and _D_-*allo*-) were separated in the second dimension by the enantioselective column. The mobile phases are the mixed solution of MeOH-MeCN containing citric acid or formic acid, and the fluorescence detection of the NBD-amino acids was carried out at 530 nm with excitation at 470 nm. All the quantitative data were obtained by the fluorescence detection.

### Data processing and multivariate analysis

Metabolites that were detected more than 50% of the samples were minimally imputed and subjected to the analysis. Data were median-centered, Pareto-scaled, and log-transformed before primary component analyses.

The association between chiral amino acids and clinical parameters was analyzed using either unsupervised principal component analysis (PCA)^12^ or supervised orthogonal partial least squares (OPLS) analysis. PCA geometrically projects complexed, higher-dimensional data onto lower dimensions called principal components. The loading plot visualizes how strongly each variable affect principal components and how each variable correlate with one another. The score value of each observation is plotted on the score plot to demonstrate the clusters of observations. OPLS model was developed to examine the discriminable distributions of a variable based on predictive variables. The goodness-of-fit for the OPLS model was evaluated using R^2^Y and Q^2^Y values. R^2^Y represents the fraction of the variance of the examined variable explained by the model, while Q^2^Y represents the predictive performance of the model. The values of R^2^Y and Q^2^Y more than 0.5 indicate a good goodness of fit, and more than 20 permutation tests per an OPLS model were performed to validate the model internally. A biplot analysis was performed to graphically project the variables and the observations as vectors and points, respectively, onto a two-dimensional principal component axes to simultaneously demonstrate the clustering of observations and the correlations of the variables^16^. PCA was performed using default setting of R environment for statistical computing version 3.4.1, and rolps library of R was applied for OPLS under default setting. A biplot display was performed using the default settings of STATA statistical software version 15 (STATA Corporation, College Station, TX, USA).

### Statistics

Continuous variables are presented as medians and ranges or interquartile ranges (IQR). Categorical variables are given as ratios (%) and counts. Continuous and categorical variables were compared using Mann-Whitney *U*-test and Fisher’s exact test, respectively. Correlations were assessed using Kendall’s tau regression analyses. Statistical significance was defined as p < 0.05. Statistical analyses were performed using STATA and R.

## Supplementary information

Supplementary Information
