# Supplementary material for: D-Serine reflects kidney function and diseases
Source: Sci Rep. 2019 Mar 25;9:5104. doi: 10.1038/s41598-019-41608-0 (PMC6434045; doi:10.1038/s41598-019-41608-0)
Supplement: Supplementary file 1 — Supplementary Information [file 41598_2019_41608_MOESM1_ESM.pdf]

Supplementary Information

D-Serine reflects kidney function and diseases

Atsushi Hesaka, Shinsuke Sakai, Kenji Hamase, Tatsuhiko Ikeda, Rakan Matsui, Masashi Mita, Masaru Horio, Yoshitaka Isaka, and Tomonori Kimura

4 Supplementary Tables
8 Supplementary Figures

Table S1. Baseline characteristics of the participants.

	Non-CKD (n = 15)	CKD (n = 11)	*P*
Age, yr	44 (39 - 50)	50 (40 - 65)	0.232
Male gender, %	80 (12)	45.5 (5)	0.103
Height, m	1.70 (1.68 - 1.75)	1.63 (1.59 - 1.66)	0.043
Weight, kg	68.9 (61.0 - 73.5)	59.8 (51.5 - 66.7)	0.194
BSA, m^2	1.80 (1.72 - 1.90)	1.61 (1.54 - 1.75)	0.102
BMI, kg / m^2	22.6 (21.1 - 25.7)	22.5 (19.3 - 24.2)	0.452
Serum creatinine, mg / dL	0.75 (0.68 - 0.83)	1.14 (0.75 - 2.59)	0.069
Serum cystatin C, mg / L	0.78 (0.69 - 0.84)	1.14 (0.87 - 2.11)	0.005
Inulin clearance, mL / min / 1.73m^2	97.0 (94.1 - 107.3)	46.0 (19.8 - 66.9)	<0.001
$eGFR_{creat}$, mL / min / 1.73m^2	87.3 (79.3 - 93.5)	49.9 (21.7 - 89.2)	0.052
$eGFR_{cys}$, mL / min / 1.73m^2	101.9 (96.4 - 114.8)	63.7 (31.8 - 94.2)	0.014

Values are described as median (IQR) or % (count). eGFR, estimated glomerular filtration ratio. *P* values, Mann-Whitney *U*-test and Fisher's exact test.

Table S2. Plasma levels of chiral amino acids.

Amino acid	Non-CKD			CKD			*P*		
	D-	L-	%D	D-	L-	%D/L	D-	L-	%D
His	ND	63.6 (57.3 - 78.6)	ND	ND	84.1 (76.9 - 96.8)	ND		0.03	
Asn	ND	39.8 (36.0 - 41.4)	ND	ND (ND - 0.63)	51.3 (46.7 - 53.6)	ND (ND - 1.21)	0.09	<0.01	0.09
Ser	1.56 (1.15 - 1.87)	93.7 (89.6 - 112.6)	1.46 (1.22 - 1.85)	2.39 (1.55 - 6.08)	114.7 (106.0 - 123.6)	1.92 (1.26 - 5.56)	0.04	0.08	0.20
Gln	ND	498.2 (431.7 - 562.3)	ND	ND	496.7 (443.4 - 562.3)	ND		0.94	
Arg	ND	62.8 (53.6 - 69.0)	ND	ND	26.0 (21.0 - 31.9)	ND		<0.01	
Asp	ND	2.48 (1.84 - 3.64)	ND	ND	4.24 (3.64 - 4.80)	ND		0.01	
Gly	-	184.0 (170.6 - 207.4)	ND	-	213.4 (182.0 - 286.2)	-		0.14	
allo-Thr	ND	ND	ND	ND	ND	ND	0.24		
Glu	ND	27.1 (21.0 - 41.7)	ND	ND	44.4 (38.4 - 64.7)	ND		0.01	
Thr	ND	108.5 (86.5 - 117.8)	ND	ND	125.6 (105.3 - 135.9)	ND		0.08	
Ala	0.99 (0.71 - 1.82)	286.7 (255.6 - 368.0)	0.30 (0.20 - 0.52)	1.28 (0.74 - 2.86)	328.5 (309.3 - 457.2)	0.37 (0.25 - 0.93)	0.42	0.14	0.78
Pro	0.48 (0.33 - 0.62)	123.2 (105.1 - 159.1)	0.34 (0.27 - 0.47)	0.78 (0.50 - 1.57)	143.1 (140.0 - 201.0)	0.44 (0.36 - 0.92)	0.03	0.02	0.07
Met	ND	22.0 (17.7 - 23.8)	ND	ND	25.6 (22.5 - 27.7)	ND		0.11	
Val	ND	230.6 (199.5 - 254.6)	ND	ND	225.4 (199.3 - 254.5)	ND		0.74	
allo-Ile	ND	ND	ND	ND	ND	ND			
Ile	ND	66.4 (53.3 - 72.4)	ND	ND	61.9 (59.3 - 64.5)	ND		0.66	
Leu	ND	132.4 (109.5 - 141.7)	ND	ND	126.4 (102.1 - 135.9)	ND	.	0.42	
Phe	ND	52.6 (48.4 - 57.9)	ND	ND	62.7 (54.5 - 66.1)	ND		0.05	
Trp	ND	ND (ND - 50.8)	ND	ND	51.2 (33.0 - 55.4)	ND		0.05	
Lys	ND	142.6 (136.3 - 154.3)	ND	ND	174.3 (147.3 - 190.3)	ND		0.05	
CysCys	ND	29.3 (25.5 - 33.8)	ND	ND	32.9 (30.1 - 53.3)	ND		0.02	
Tyr	ND	52.1 (50.0 - 66.7)	ND	ND	58.2 (49.4 - 68.7)	ND		0.98	

Values, μM; ND, not determined; %D, D-amino acids per total amino acids. *P* values, Mann-Whitney *U*-test between non-CKD and CKD groups.

Table S3. Urinary levels of chiral amino acids.

Amino acid	Non-CKD D-	Non-CKD L-	Non-CKD %D	CKD D-	CKD L-	CKD %D	*P* D-	*P* L-	*P* %D
His	ND	671.7 (582.3 - 806.0)	ND	ND (ND - 1.16)	524.5 (237.9 - 1104.9)	ND (ND - 0.17)	0.09	0.48	0.09
Asn	13.2 (10.3 - 14.5)	53.3 (35.8 - 64.2)	18.4 (13.0 - 24.8)	12.1 (8.46 - 16.4)	65.8 (22.1 - 209.3)	13.6 (3.52 - 33.5)	0.62	0.52	0.59
Ser	123.4 (108.7 - 131.8)	144.7 (97.8 - 230.3)	45.4 (32.6 - 56.2)	112.0 (101.2 - 151.8)	202.4 (71.8 - 430.4)	36.3 (27.2 - 58.5)	0.90	0.66	0.98
Gln	1.90 (ND - 2.60)	326.9 (249.7 - 422.4)	0.55 (ND - 0.75)	2.80 (2.45 - 3.62)	335.3 (228.9 - 1166.4)	0.66 (0.34 - 1.29)	0.01	0.55	0.16
Arg	ND	ND	ND	ND	18.2 (5.5 - 53.1)	ND		0.01	
Asp	ND	5.20 (3.82 - 7.35)	ND	ND	5.5 (1.7 - 10.8)	ND	.	0.79	
Gly	-	823.9 (571.8 - 998.0)	-	-	965.2 (462.2 - 2363.9)	-		0.62	
allo-Thr	7.10 (6.20 - 8.37)	ND	100 (100 - 100)	8.17 (6.06 - 8.87)	ND	100 (100 - 100)	0.80		0.98
Glu	ND	10.0 (7.8 - 12.4)	ND	ND (ND - 1.83)	16.6 (11.2 - 36.3)	ND (ND - 10.7)	0.05	0.02	0.39
Thr	0.53 (ND - 1.39)	98.3 (70.8 - 134.2)	0.45 (ND - 1.27)	1.33 (0.94 - 1.79)	133.2 (57.3 - 301.0)	0.64 (0.19 - 1.40)	0.10	0.42	0.35
Ala	28.4 (23.1 - 41.7)	138.4 (74.7 - 209.9)	12.2 (10.7 - 28.0)	25.2 (14.7 - 42.3)	214.9 (103.4 - 511.3)	12.7 (6.98 - 14.9)	0.66	0.24	0.19
Pro	ND	3.89 (3.64 - 5.19)	ND	ND (ND - 0.70)	12.2 (7.2 - 18.5)	ND (ND - 0.27)	0.09	0.00	0.09
Met	ND	ND (ND - 7.6)	ND	ND	5.9 (1.6 - 11.9)	ND		0.17	
Val	ND	28.8 (22.8 - 38.5)	ND	0.38 (ND - 0.46)	37.7 (22.3 - 84.9)	0.02 (ND - 1.06)	0.00	0.52	0.03
allo-Ile	ND	ND	ND	ND	ND	ND	0.09		0.09
Ile	ND	8.42 (7.30 - 11.5)	ND	ND	11.0 (6.4 - 16.9)	ND		0.59	
Leu	ND	20.0 (17.2 - 32.7)	ND	0.36 (ND - 0.65)	28.6 (14.5 - 54.4)	0.14 (ND - 1.69)	0.01	0.59	0.10
Phe	ND	40.2 (26.0 - 51.4)	ND	ND (ND - 0.49)	49.6 (21.7 - 77.8)	ND (ND - 0.94)	0.01	0.59	0.17
Trp	ND	ND (ND - 61.9)	ND	ND	ND	ND		0.74	
Lys	ND	119.5 (93.9 - 212.8)	ND	ND (ND - 3.26)	122.9 (46.9 - 668.7)	ND (ND - 0.81)	0.06	0.82	0.43
CysCys	ND	39.2 (14.9 - 59.2)	ND	ND	103.5 (34.2 - 304.4)	ND		0.17	
Tyr	ND	80.4 (54.7 - 100.8)	ND	ND	76.2 (15.1 - 97.8)	ND		0.66	

Values, moL / gram creatinine (gCre); %D, D-amino acids per total amino acids; ND, not determined. *P* values, Mann-Whitney *U*-test.

Table S4. Fractional excretions of chiral amino acids.

Amino acid	Non-CKD		CKD		*P*	
	D-	L-	D-	L-	D-	L-
His	ND	7.55 (5.29 - 10.73)	ND	7.96 (2.19 - 16.70)		0.86
Asn	ND	1.00 (0.73 - 1.33)	ND	1.12 (0.48 - 10.52)	0.24	0.74
Ser	62.1 (53.4 - 73.6)	1.30 (0.75 - 1.96)	50.5 (47.0 - 72.5)	1.21 (0.52 - 9.70)	0.45	0.82
Gln	ND	0.53 (0.35 - 0.76)	ND	0.70 (0.30 - 4.36)		0.45
Arg	ND	ND (ND - 0.04)	ND	0.38 (0.17 - 2.28)		<0.01
Asp	ND	1.40 (0.88 - 2.79)	ND	1.15 (0.29 - 5.54)		0.96
Gly	ND	3.42 (2.25 - 3.81)	ND	4.39 (2.41 - 17.12)		0.29
allo-Thr	ND	ND	ND	ND		
Glu	ND	0.28 (0.199 - 0.37)	ND	0.26 (0.23 - 1.41)		0.78
Thr	ND	0.70 (0.47 - 0.98)	ND	0.98 (0.47 - 7.57)		0.39
Ala	20.7 (17.7 - 22.2)	0.28 (0.21 - 0.44)	22.8 (18.3 - 39.4)	0.40 (0.26 - 2.71)	0.20	0.26
Pro	ND	0.026 (0.022 - 0.031)	ND (ND - 1.7)	0.056 (0.041 - 0.25)	0.09	<0.01
Met	ND	0.00 (0.00 - 0.22)	ND	0.19 (0.050 - 0.38)		0.23
Val	ND	0.099 (0.077 - 0.12)	ND	0.16 (0.077 - 0.71)		0.11
allo-Ile	ND	ND	ND	ND		
Ile	ND	0.097 (0.088 - 0.14)	ND	0.17 (0.084 - 0.38)		0.19
Leu	ND	0.12 (0.114 - 0.18)	ND	0.23 (0.11 - 0.58)		0.14
Phe	ND	0.48 (0.39 - 0.66)	ND	0.70 (0.33 - 2.45)		0.39
Trp	ND	ND (ND - 0.00)	ND	ND (ND - 1.27)		0.05
Lys	ND	0.71 (0.46 - 1.07)	ND	0.92 (0.32 - 5.31)		0.66
CysCys	ND	1.05 (0.34 - 1.41)	ND	2.58 (1.02 - 9.38)		0.11
Tyr	ND	1.03 (0.72 - 1.27)	ND	0.99 (0.18 - 3.59)		0.81

Values, %; ND, not determined. *P* values, Mann-Whitney *U*-test.

Figure S1

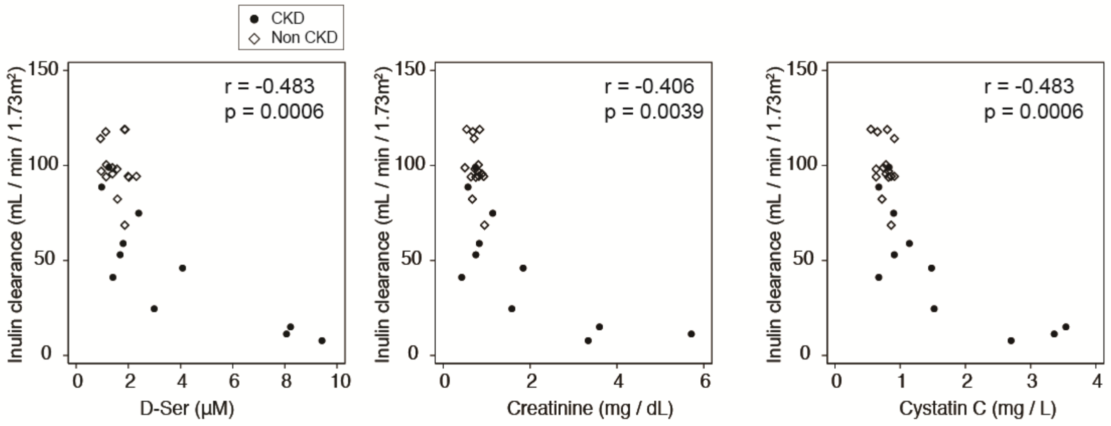

Figure S1. Relation of plasma D-serine and GFR. Blood non-log transformed levels of D-serine, creatinine, and cystatin C are plotted with GFR (mL / min / 1.73m^2). Correlations, Kendall's tau regression analyses.

Figure S2

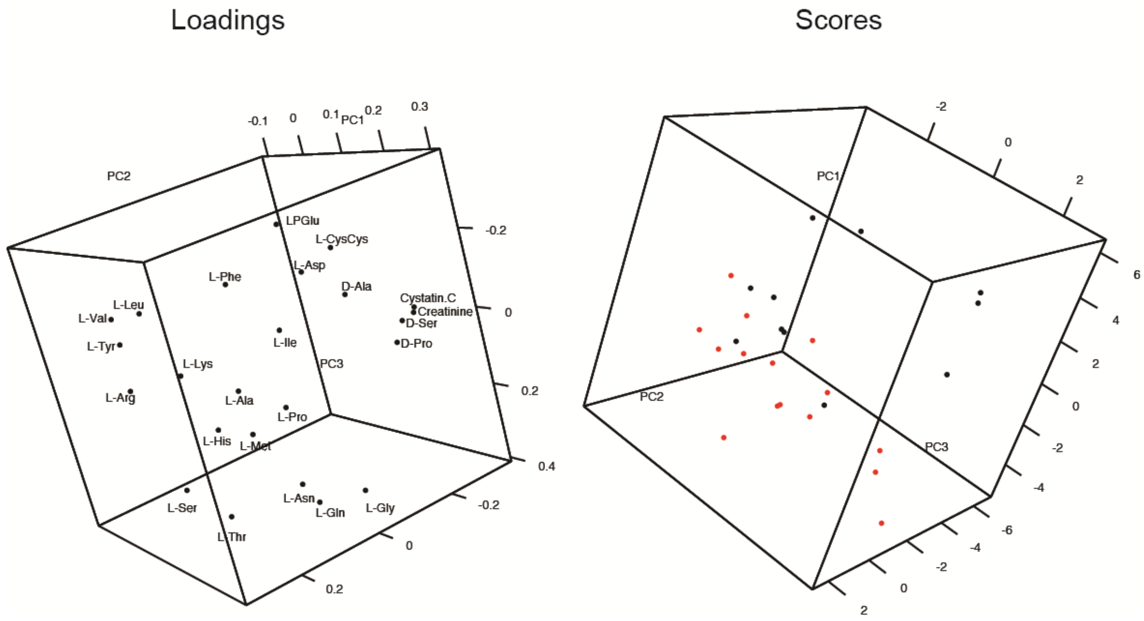

Figure S2. PCA of plasma chiral amino acids and kidney markers. Red and black dots in the score plot denote non-CKD and CKD participants, respectively. Although the cumulative portions for both 2D and 3D analyses were high (87% and 91%, respectively), we displayed 3D analysis because formations of clusters were best seen in 3D in our view. The score plot shows the clustering of the presence or absence of CKD achieved by this analysis.

Figure S3

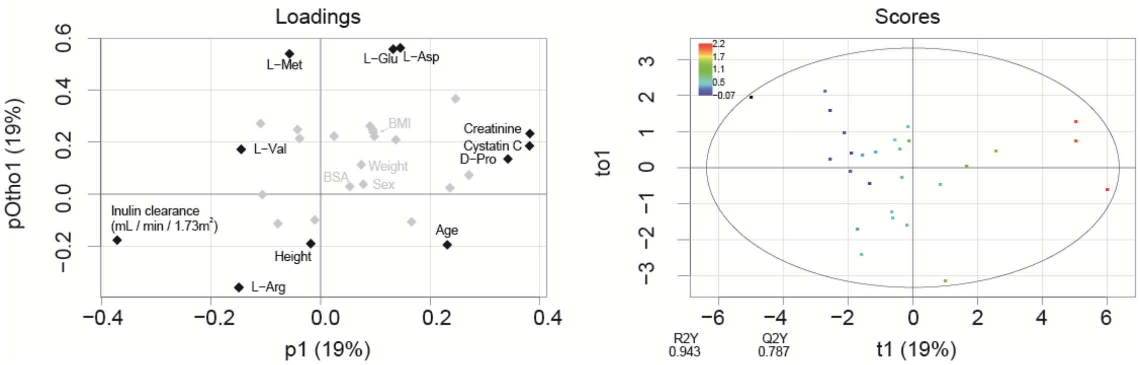

Figure S3. Relation of D-serine dynamics and disease profile. OPLS derived from the chiral amino acid profile, serum levels of creatinine and cystatin C, and clinical factors on GFR. Clinical factors used in this model are indicated. The remaining of gray symbols represent plasma L-amino acids. The score plot is colored according to LN GFR. The circle in the score plot represents the 95% confidence interval.

Figure S4

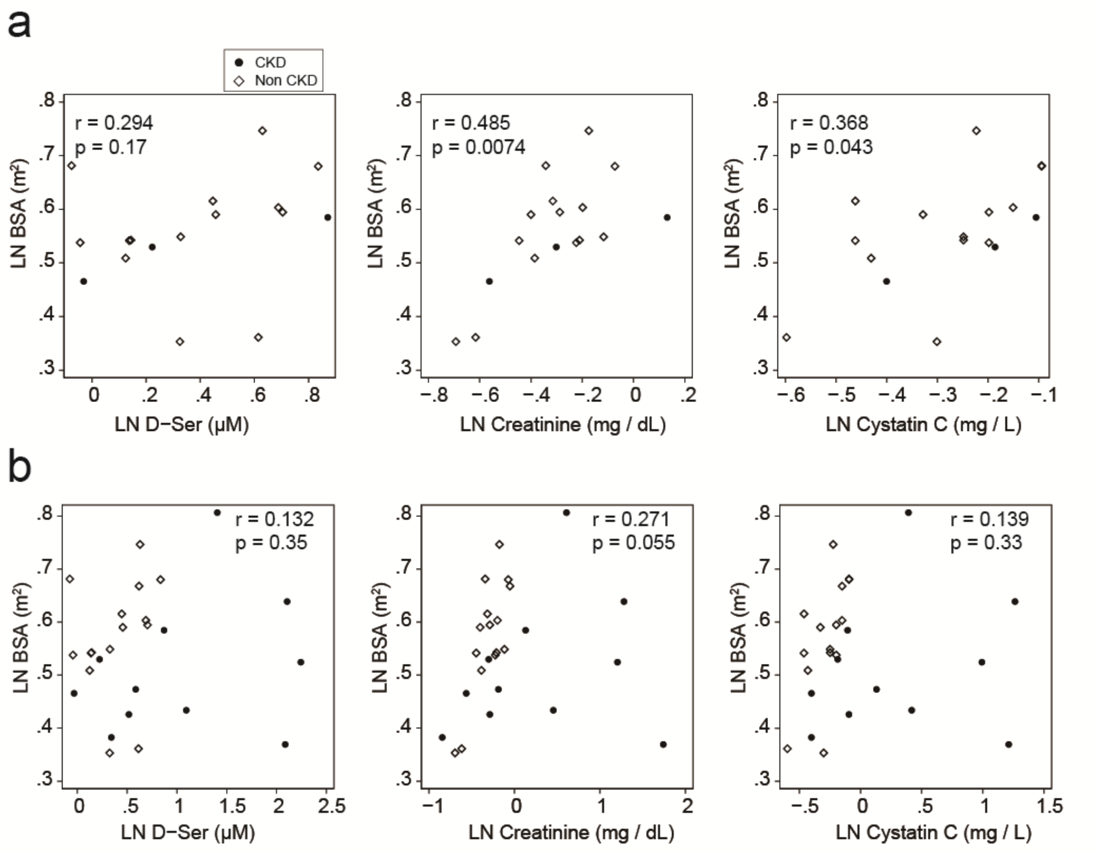

Figure S4. Relation of plasma D-serine and body surface area (BSA). (a-b) Blood levels of D-serine, creatinine, and cystatin C are plotted with BSA (m^2) in (a) participants with GFR > 70 mL / min / 1.73m^2, or in (b) whole participants. Correlations, Kendall's tau regression analyses.

Figure S5

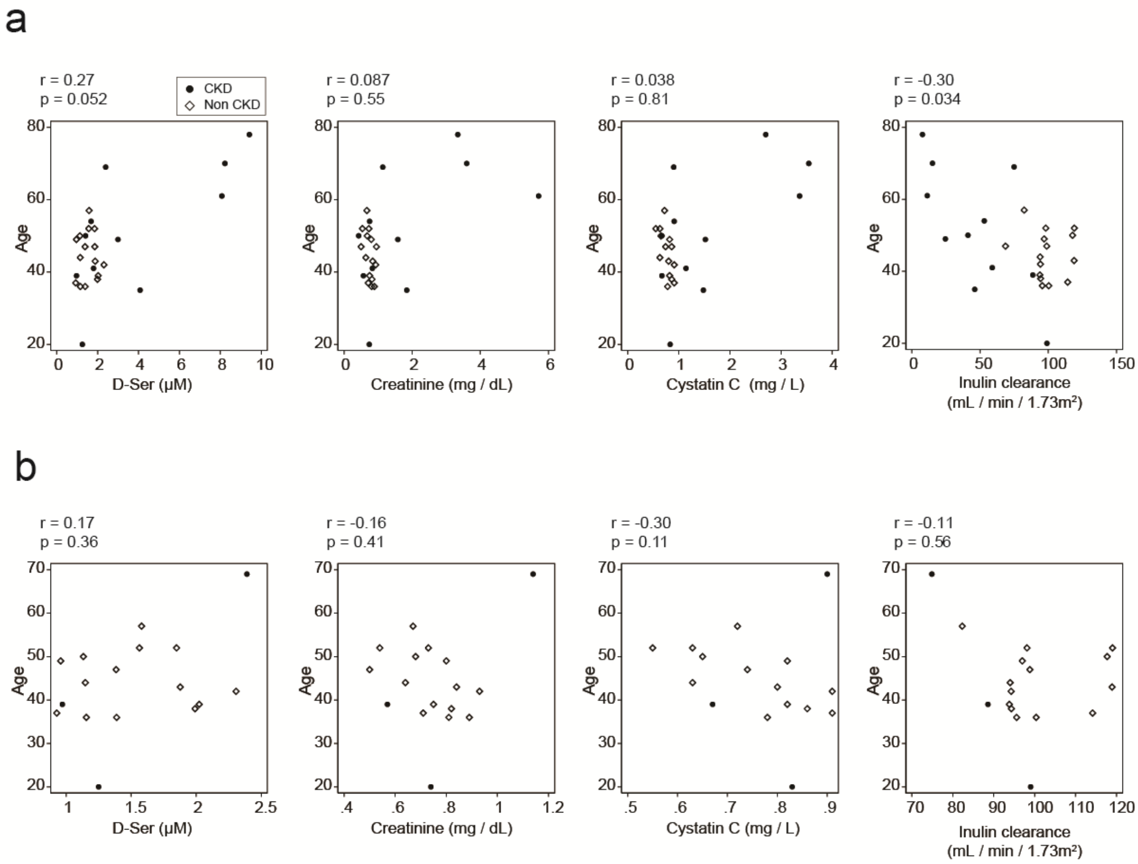

Figure S5. Relation of plasma D-serine and age. (a-b) Blood levels of D-serine, creatinine, and cystatin C, or GFR (inulin clearance, mL / min / 1.73m^2), are plotted with age in (a) whole participants, or in (b) participants with GFR > 70 mL / min / 1.73m^2. Correlations, Kendall's tau regression analyses.

Figure S6

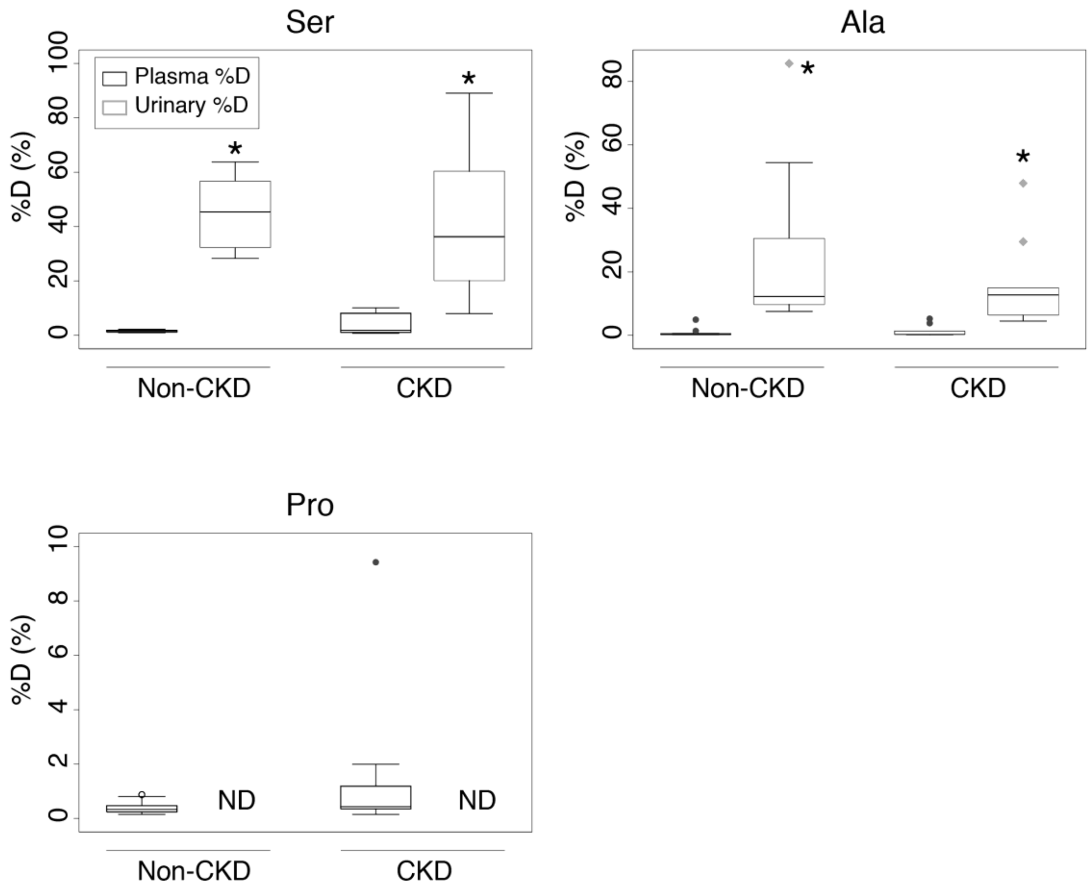

Figure S6. Characteristics of chiral amino acids ratios. Box plots of D-amino acids per total amino acids (%D) in plasma and in urine. ND, not determined. *$P < 0.05$ between plasma and urinary %D in each group (Mann-Whitney *U*-test).

Figure S7

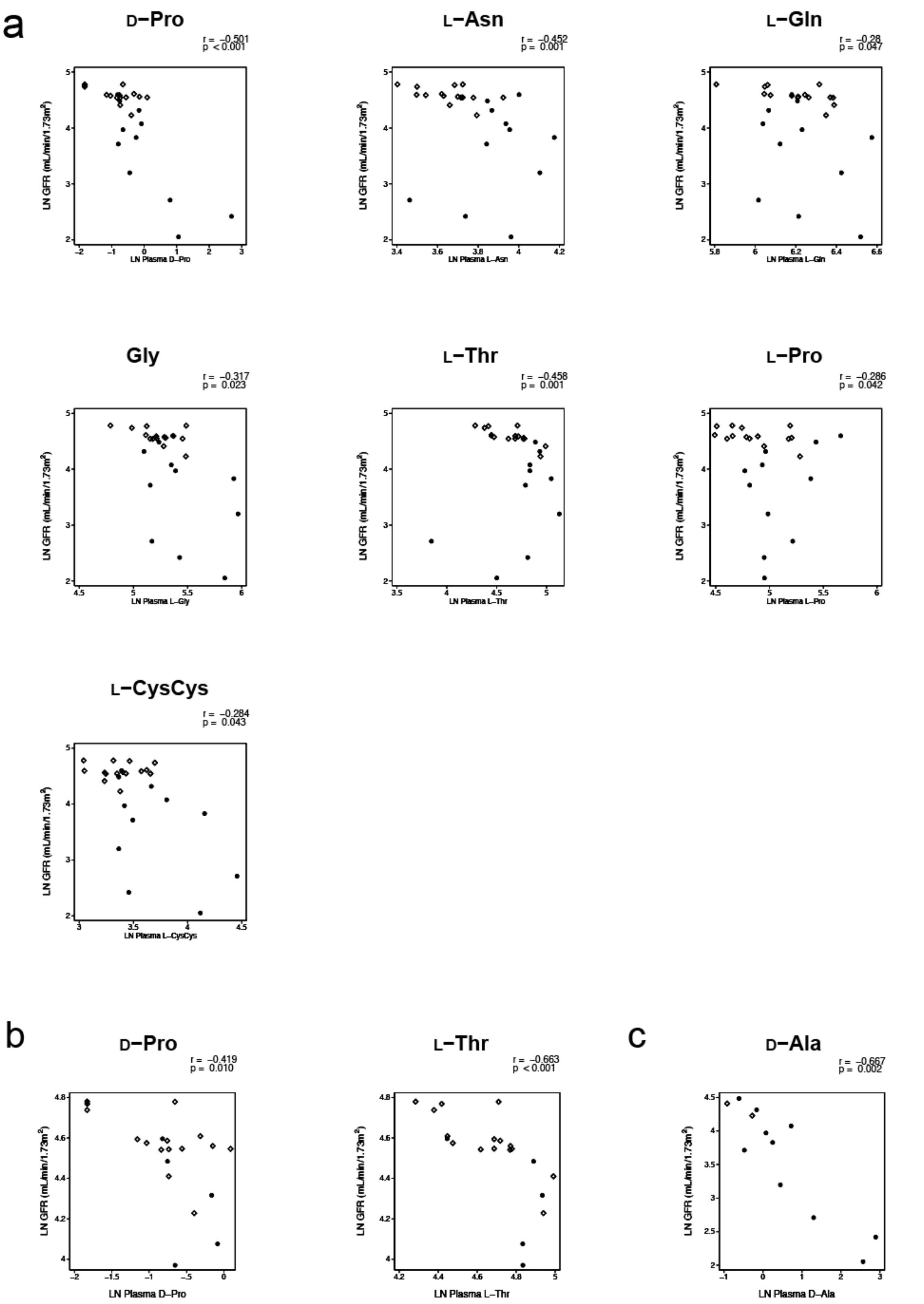

Figure S7. Relation of plasma chiral amino acids and GFR. (a-c) Plasma levels

of log-transformed chiral amino acids are plotted with GFR (mL / min / 1.73m^2) in (a) whole participants, and in participants with (b) GFR > 50 mL / min / 1.73m^2 or (c) GFR < 90 mL / min / 1.73m^2. Correlations, Kendall's tau regression analyses.

Figure S8

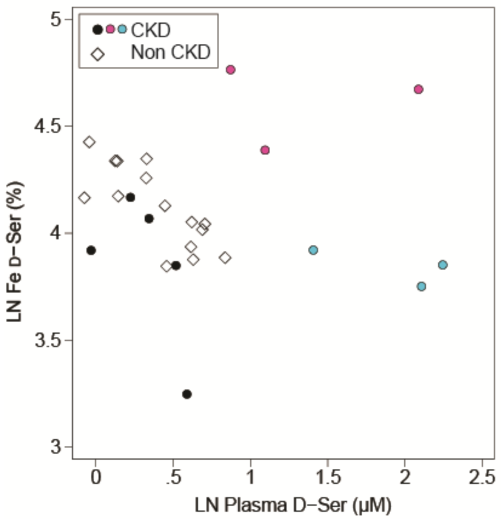

Figure S8. Relation of D-serine dynamics and disease profile. The relation shown in Figure 3b is highlighted as discussed. Magenta, CKD patients with increased Fe of D-serine. Cyan, CKD patients with increased blood levels of D-serine and no increase in Fe.
